# Tracking the impacts of climate change on human health via indicators: lessons from the *Lancet* Countdown

**DOI:** 10.1186/s12889-022-13055-6

**Published:** 2022-04-06

**Authors:** Claudia Di Napoli, Alice McGushin, Marina Romanello, Sonja Ayeb-Karlsson, Wenjia Cai, Jonathan Chambers, Shouro Dasgupta, Luis E. Escobar, Ilan Kelman, Tord Kjellstrom, Dominic Kniveton, Yang Liu, Zhao Liu, Rachel Lowe, Jaime Martinez-Urtaza, Celia McMichael, Maziar Moradi-Lakeh, Kris A. Murray, Mahnaz Rabbaniha, Jan C. Semenza, Liuhua Shi, Meisam Tabatabaei, Joaquin A. Trinanes, Bryan N. Vu, Chloe Brimicombe, Elizabeth J. Robinson

**Affiliations:** 1 School of Agriculture, Policy and Development, University of Reading, Reading, UK; 2 Department of Geography and Environmental Science, University of Reading, Reading, UK; 3 Institute for Global Health, University College London, London, UK; 4 Institute for Risk and Disaster Reduction, University College London, London, UK; 5 School of Global Studies, University of Sussex, Brighton Falmer, UK; 6 United Nations University, Institute for Environment and Human Security, Bonn, Germany; 7 Ministry of Education Key Laboratory for Earth System modeling, Department of Earth System Science, Tsinghua University, Beijing, 100084 China; 8 Institute for Environmental Science, University of Geneva, Geneva, Switzerland; 9 Grantham Research Institute on Climate Change and the Environment, London School of Economics and Political Science (LSE), London, UK; 10 Centro Euro-Mediterraneo sui Cambiamenti Climatici (CMCC), Venice, Italy; 11 Università Ca’ Foscari, Venice, Italy; 12 Department of Fish and Wildlife Conservation, Virginia Tech, Blacksburg, VA USA; 13 University of Agder, Kristiansand, Norway; 14 Health and Environment International Trust, Nelson, New Zealand; 15 Rollins School of Public Health, Emory University, Atlanta, USA; 16 Barcelona Supercomputing Center, Barcelona, Spain; 17 Catalan Institution for Research and Advanced Studies (ICREA), Barcelona, Spain; 18 Centre on Climate Change & Planetary Health and Centre for Mathematical Modelling of Infectious Diseases, London School of Hygiene & Tropical Medicine, London, UK; 19 Department of Genetics and Microbiology, Universitat Autònoma de Barcelona (UAB), Barcelona, Spain; 20 School of Geography, Earth and Atmospheric Sciences, The University of Melbourne, Melbourne, Australia; 21 Preventive Medicine and Public Health Research Center, Psychosocial Health Research Institute, Iran University of Medical Sciences, Tehran, Iran; 22 MRC Centre for Global Infectious Disease Analysis, Imperial College London, London, UK; 23 MRC Unit The Gambia At London School of Hygiene and Tropical Medicine, Atlantic Boulevard, Fajara, The Gambia; 24 Iranian Fisheries Science Research Institute, Agricultural Research, Education, and Extension Organisation, Tehran, Iran; 25 Heidelberg Institute of Global Health, University of Heidelberg, Heidelberg, Germany; 26 Higher Institution Centre of Excellence (HICoE), Institute of Tropical Aquaculture and Fisheries (AKUATROP), Universiti Malaysia Terengganu, 21030 Kuala Nerus, Terengganu Malaysia; 27 Henan Province Forest Resources Sustainable Development and High-value Utilization Engineering Research Center, School of Forestry, Henan Agricultural University, Zhengzhou, 450002 China; 28 Department of Electronics and Computer Science, Universidade de Santiago de Compostela, Santiago, Spain

**Keywords:** Climate change, Public health, Indicators, Climate data, Policy making

## Abstract

**Background:**

In the past decades, climate change has been impacting human lives and health via extreme weather and climate events and alterations in labour capacity, food security, and the prevalence and geographical distribution of infectious diseases across the globe. Climate change and health indicators (CCHIs) are workable tools designed to capture the complex set of interdependent interactions through which climate change is affecting human health. Since 2015, a novel sub-set of CCHIs, focusing on climate change impacts, exposures, and vulnerability indicators (CCIEVIs) has been developed, refined, and integrated by Working Group 1 of the “*Lancet* Countdown: Tracking Progress on Health and Climate Change”, an international collaboration across disciplines that include climate, geography, epidemiology, occupation health, and economics.

**Discussion:**

This research in practice article is a reflective narrative documenting how we have developed CCIEVIs as a discrete set of quantifiable indicators that are updated annually to provide the most recent picture of climate change’s impacts on human health. In our experience, the main challenge was to define globally relevant indicators that also have local relevance and as such can support decision making across multiple spatial scales. We found a hazard, exposure, and vulnerability framework to be effective in this regard. We here describe how we used such a framework to define CCIEVIs based on both data availability and the indicators’ relevance to climate change and human health. We also report on how CCIEVIs have been improved and added to, detailing the underlying data and methods, and in doing so provide the defining quality criteria for *Lancet* Countdown CCIEVIs.

**Conclusions:**

Our experience shows that CCIEVIs can effectively contribute to a world-wide monitoring system that aims to track, communicate, and harness evidence on climate-induced health impacts towards effective intervention strategies. An ongoing challenge is how to improve CCIEVIs so that the description of the linkages between climate change and human health can become more and more comprehensive.

## Background

Climate change affects global health via multiple direct and indirect pathways [1, 2]. Every year, disasters involving weather- and climate-related hazards result in thousands of deaths worldwide and contribute to the global burden of disease [3, 4]. Direct health consequences may derive from changes in temperature and precipitation, and human exposure to heatwaves, wildfires, floods, and droughts [5–9]. These include an increase in cardiovascular mortality during events of extreme heat; a higher incidence of chronic kidney disease among outdoor workers in hot areas; and fatalities and multiple negative health consequences of fire smoke inhalation during wildfires [10–13]. Indirect impacts may be triggered by climate change-induced environmental and ecosystem alterations, such as crop failures, reduced marine food capture, geographic range expansion of disease vectors, and reduced labour capacity [14–17]. Other indirect effects may be mediated through social systems and responses to climate change. Examples include reduced labour capacity in vulnerable occupations due to increasing heat, migration of populations related to sea-level rise and food insecurity, as well as other drivers of human mobility [18–20].

As understanding and awareness grow regarding the health dimension of climate change, there is an increased demand for feasible and efficient tools that can inform climate change mitigation and adaptation strategies to deliver benefits to human health at all levels. Climate-health linkages are complex, with multiple interactions, synergies, and feedback loops. Understanding their characteristics and relationships is challenging and poses a level of complexity that could render the development of such tools unfeasible. A reasonable alternative is to deploy a subset of measures describing the key aspects of the state, extent and change of the climate-health system [21].

Climate change and health indicators (CCHIs) are summary measures, often quantitative, that represent the heterogeneous set of factors and relationships linking climate change with health [22–26]. Their purpose is three-fold. First, to assess long-term trends and changes in climate-induced impacts on population health. Second, to effectively communicate relevant evidence to researchers, health professionals, policymakers, and the general public. Third, to support and monitor decision making for successful intervention strategies and plans that address the human health consequences of climate change.

In the past decade, CCHIs have represented the building blocks for a variety of initiatives tracking the climate-health system across multiple spatial and temporal scales [24, 25]. One high profile and current CCHI initiative is the “*Lancet* Countdown: Tracking Progress on Health and Climate Change”. Since 2015 the *Lancet* Countdown has been providing a global monitoring system that tracks the complex, reflective ways in which climate change affects health [27–32]. It accomplishes this through a set of CCHIs spanning five domains of the climate-health system: climate change impacts, exposures, and vulnerability; adaptation planning and resilience for health; mitigation actions and health co-benefits; economics and finance; and public and political engagement.

One of the five *Lancet* Countdown thematic working groups, namely Working Group 1 (WG1), is tasked with identifying and tracking indicators that trace the links across “climate change impacts, exposures, and vulnerability”. This research in practice article aims to provide guidance for the development and tracking of CCHIs through a detailed case study of how WG1 authors developed these indicators, referred to from here on as *climate change impacts, exposures, and vulnerability indicators* (CCIEVIs), that contribute to the *Lancet* Countdown’s overall objectives. As such, this article is equally relevant for those individuals and policy makers wanting to develop their own climate change and health indicators specific to a country or region, and those wanting a more detailed understanding of specific indicators used in the *Lancet* Countdown, and particularly WG1. Hereafter, we describe (i) the aim, design, and characteristics of CCIEVIs, as employed in the *Lancet* Countdown, (ii) their main components, (iii) underlying data and methods, and (iv) plans for future improvements.

## Climate change impacts, exposures, and vulnerability indicators

### Designing CCIEVIs

*Lancet* Countdown CCIEVIs have been developed to monitor the health impacts related to anthropogenic climate change, their trends to date, and the extent to which progress (or backsliding) is occurring over time. They are presented from a historical starting date up to the year for which the most recent complete data are available. The indicators provide evidence along different dimensions – some indicators are positioned closer to the climate change hazard and some closer to the health impact – as to whether, and to what extent, climate change is affecting health, for better or for worse. In the *Lancet* Countdown, CCIEVIs start tracking climate change-health linkages from the 1980s, and are grouped into five thematic clusters: heat; weather and climate extremes; infectious diseases; food security of terrestrial and marine assets; global mean sea-level rise.

To ensure that CCIEVIs are reproducible, relevant, and useful to scientific and policy-making communities, they must satisfy five quality criteria that mirror the World Meteorological Organisation guidelines for climate indicators [33]:

1. *Representativeness:* a CCIEVI should track an aspect of both climate change and health, particularly focusing on the relationship between the two; it should do this across a timescale and geographical coverage sufficient for long-term global trends to be observed.
2. *Relevance:* a CCIEVI should be clear and understandable to a broad range of audiences; global CCIEVIs may also have value at local (i.e. national, regional) level for policy and decision makers.
3. *Robustness:* a CCIEVI should use data and methods that are robust, reliable, and valid to track the relevant aspect of climate change and health; data from publicly available databases, and especially those developed by international organisations, governmental bodies, or academic institutions, are preferred; methods should be supported by a high standard of evidence from the scientific literature.
4. *Reproducibility:* a CCIEVI should be calculated using an internationally agreed and published scientific method as well as open-access and quality-controlled data; the methodology underlying an indicator should be clearly laid out, including details of the process of data collection and processing, which must be done in a systematic and unbiased way; statistical analysis of the data should be carried out to support the interpretation of the data.
5. *Timeliness:* a CCIEVI should be calculated regularly, with a short lag between the end of the period under consideration and the publication of the data; the calculation must be practicable with existing and future resources.

WG1 indicators have evolved over the five years that they have been reported in the annual *Lancet* Countdown publications so to fulfil above-listed criteria. For example, in 2020, there were twelve WG1 *Lancet* Countdown CCIEVIs, almost double the number of when they first appeared in 2016 [28, 32]. As well as new indicators being introduced each year, established indicators are revised to reflect the latest evidence in the scientific literature and the needs of new and emerging stakeholders [30, 31]. The revision of *Lancet* Countdown CCIEVIs occurs annually by experts across a broad range of relevant disciplines, including climate, geography, epidemiology, and occupation health. The publication of an appendix alongside each *Lancet* Countdown annual report ensures that the data and methods underlying every CCIEVI are explicitly cited and described along with possible caveats.

### Main components of CCIEVIs

*Lancet* Countdown CCIEVIs focus on three main components: hazard, exposure, and vulnerability. These are defined according to the terminology provided by the United Nations Office for Disaster Risk Reduction and the Intergovernmental Panel on Climate Change [34, 35]. Specifically:

- Hazard is any physical event with the potential to cause disruption or damage in vulnerable and exposed elements;
- Exposure is who or what is present in the area where a hazard may occur;
- Vulnerability is the factors or constraints of an economic, social, physical, or geographic nature, which reduce the ability of exposed elements to prepare for and cope with the impact of the hazard.

In *Lancet* Countdown CCIEVIs, each of these components represents a dimension or *layer* of the multi-facet climate-health system. For the hazards, the *Lancet* Countdown CCIEVIs target extremes in temperature and precipitation, namely heatwaves, drought, and floods, as well as phenomena mediated by these two environmental variables, i.e. wildfires and suitability of vector-borne diseases (malaria, dengue). Similarly, health hazards in the ocean system are identified in changes of global mean sea level as well as sea surface temperature and salinity. The second layer tracks the exposure of populations, cultivated areas, and health systems to hazards. This reflects the purpose of representing climate change-health pathways that are both direct (e.g. on people) and indirect (e.g. food mediated). *Lancet* Countdown CCIEVIs also track vulnerabilities to extreme heat, incorporating the proportion of the population over 65 years of age, the prevalence of predisposing chronic diseases (i.e. diabetes and cardiovascular, respiratory, and renal disease), the proportion of the population living in urban areas, and the number of outdoor workers. In the 2021 report, the vulnerability of children younger than 1 year to life-threatening heatwaves was added [36]. *Lancet* Countdown CCIEVIs also consider vulnerability linked to the capacity of local health services to respond to public health risks and emergencies.

### Data and methods for developing CCIEVIs

Hazards, exposures, and vulnerabilities change over time and across space as do their interactions as a result of changes in the climate. Consequently, climate change amplifies or diminishes existing health impacts, and induces or suppresses new ones with a high degree of spatial and temporal variability. Geographic information systems, and more specifically geospatial data, have long been identified as useful analytical tools for monitoring the health-climate system at all spatial levels, from local to global [37].

Many relevant sources for CCIEVIs provide the data as rasters, i.e. grids of cells. This allows information, such as variations in climate-relevant variables like air temperature, to be represented in a spatially continuous and consistent way across the Earth’s surface. Usefully, rasters can be stacked to assess cumulative hazards in a given region or to produce spatially resolved time series of a given variable.

Environmental data are nowadays available as rasters across a wide range of spatial and temporal scales. This makes them an ideal tool for constructing relationships between hazards, exposed elements, and associated vulnerability anywhere in the world, low and middle income countries included [38]. For instance, one of the *Lancet* Countdown CCIEVIs tracks the climatic suitability for the transmission of *Plasmodium falciparum*, the parasite causing malaria. Following the work by Grover-Kopec and colleagues [39], the indicator tracks the number of months per year suitable for malaria transmission as the coincidence of precipitation accumulation greater than 80 mm, average temperature between 18 °C and 32 °C, and relative humidity greater than 60%. The number of suitable months in a year is calculated for each grid cell by *overlaying* precipitation, temperature, and humidity raster layers across twelve months. Year-by-year changes in the number of suitable months generate a time series, which can be spatially aggregated (e.g. for highland vs lowland areas).

CCIEVIs can be explored via an online visualization platform. For example, maps represent a powerful way to visualise and communicate climate information, and harness the latter for action [40]. CCIEVIs can be displayed as a set of world-wide maps that can be navigated via an interactive, user-friendly interface to show year-specific data or to highlight countries or geographical areas of interest, as has been done by the *Lancet* Countdown [41].

## Perspectives and improvements for CCIEVIs

Climate change is an evolving phenomenon and our understanding of it is also evolving. Only with the most up-to-date data, methodologies, and expertise, CCIEVIs can provide the quantitative underpinnings of a compelling narrative on the health impacts that climate change imposes across the globe. Based on our experience from the *Lancet* Countdown, we here report the main points CCIEVIs might consider to be meaningful and useful in their purpose.

1. *Partnership:* The cross-cutting nature of CCIEVIs demands a combination of skills, knowledge and data that span across institutions, disciplines, countries, and geographical regions. Creating and maintaining a long-term collaboration among a group of diverse experts is crucial to guaranteeing the robustness and reliability of developed CCIEVIs over time. Partnership can also foster the development of CCIEVI-based initiatives in locations where these are currently missing but are perceived as useful [42–46]. Over the long term, partnerships can expand the areas where CCIEVIs drive decision-making as well as increase workforce preparedness via the inclusion of climate change curriculum into health professional education [47–50].
2. *Iterative process:* To satisfy the timeliness criteria of CCIEVIs, their design and utility must be revised periodically [21]. Every year, *Lancet* Countdown CCIEVIs indicators undergo a thorough quality check and improvement process before being considered for the annual report. In this process, independent experts assess the quality and suitability of each indicator and provide constructive feedback to aid their development and improvement. Additionally, new CCIEVIs can be developed and added to the original indicators suite to provide a more complete description of the climate-health system.
3. *Going local*: Because CCIEVIs are provided as geospatial data, they are down-scalable and able to identify priority areas for public health intervention across the globe. Location-specific CCIEVIs can assist local health departments in tracking variations in community exposure and vulnerability to climate change, uncovering health impacts at regional or sub-regional level (including their linkages to the surrounding urban/natural environment), designing interventions to enhance community resilience and evaluating the effectiveness of implemented interventions [51, 52]. With this motivation, the *Lancet* Countdown CCIEVIs structure has been replicated to produce the Australia and China reports with indicators being provided at national to regional and city scales [53, 54]. These local reports may serve as a guidance for disseminating progress on CCIEVIs from other countries in a uniform format. Every year, the *Lancet* Countdown report is also followed by a range of resources, such as briefs for policymakers and translations of the executive summaries, tailored to specific cities, countries and geographical areas [55–57].
4. *Data*: CCIEVIs typically have to rely on different sources for the provision of health, climate, and demographics data. They therefore may well differ in the spatial resolution, the time period covered, and the reference baseline used for the definition of extreme events. Notwithstanding this, consistency in climate data and demographics among *Lancet* Countdown CCIEVIs has been attempted and achieved wherever possible so that indicators are compatible and comparable with each other. It is worth noting that protocols are in place for the effective archival, management, analysis, delivery, and use of climate data, whereas health data standardisation is an open topic [58, 59]. The collection and reporting of public health data, for instance, vary greatly across nations and healthcare organizations [60]. Data monitoring on disease incidence or health outcomes that are standardised at a global level is in general lacking. In most cases, health impacts are therefore tracked using epidemiological models rather than measured records. Furthermore, their geographical resolution is generally lower than climate data (i.e. country level rather than at raster cell level). Unlike climate data, health records are generally not publicly available. Creating open-access online databases for public health data would help foster knowledge exchange between the climate and the public health community, as well as promote its applicability at all scales.

CCIEVIs can be used as a starting point for broader explorations of the linkages between climate change and health that have relevance for policy making. Here we suggest six specific areas for future research, many of which are currently being pursued by WG1 authors.

1. *Looking to the future:* In the *Lancet* Countdown, CCIEVIs are used to track the current health impacts of climate change, and contextualise them with respect to the recent past. These indicators therefore hint at how the future will likely evolve without efforts to mitigate and adapt to climate change. However, the methodology described in this research in practice article is time-agnostic and can be applied to indicators predictive of the future. Designing, developing and implementing future projections of CCIEVIs presents additional research challenges, such as the choice of the climate prediction model and its verification, but has the potential to provide actionable information to support policymaking [61].
2. *Identifying hotspots*: Climate change often affects people and places in multiple ways. We define *climate-health hotspots* as locations where climate affects people negatively through multiple pathways. This definition builds upon work already established in the climatology field on mapping *hotspots*, i.e. geographical areas where the combined occurrence of multiple weather extremes (compound events) is observed [62, 63]. As adverse health effects occur in populations that are already at risk from climate extremes and lack adequate health infrastructure [64–66], the overlay method described in this work can be adapted to identify climate-health hotspots and trace them overtime by overlapping multiple layers of hazard, exposure and vulnerability. It can also be applied to different CCIEVIs to assess multiple climate change impacts in a single location. This could allow for a *one health* perspective if CCIEVIs at the human–animal–environment interface are implemented [67, 68]. For instance, concerning food security of terrestrial assets, a CCIEVI tracking changes in crop yield potential due to rising temperatures could be overlayed with CCIEVIs monitoring animal-source foods and/or the effects of environmental changes on diseases of livestock and crops. In either case, the identification and tracking of climate-health hotspots and incorporation into a monitoring system, could improve further public health action, particularly in the context of reducing the likelihood of systemic health crises [69].
3. *Inequality and inequity:* Global indicators generally do not provide a nuanced picture of the differentiated impacts of climate change on health by country and region, or across populations within countries. Disaggregating indicators by relevant socioeconomic and occupational characteristics (e.g. income, gender, race or ethnicity, disability, occupation, and age) is necessary to measure inequalities in exposure to climatic risk factors and health outcomes attributable to these exposures. For heatwaves, for instance, these may consider pregnancy and mental health [70, 71]. With this approach, climate change and climate-related risk factors are positioned as both environmental and social determinants of health.
4. *Mental health:* Mental health issues are closely intertwined with people’s geographical, social, environmental, financial, and political context, and any climate-induced impact on physical health is also a risk factor for mental health [72]. As such, factors linking climate change to health may affect mental health and underlying mental health status can affect the capacity to adapt to (or to mitigate) climate change. The description of the climate-health system, therefore, requires the inclusion of CCIEVIs that address the mental health consequences of climate change. A growing body of literature is currently examining these linkages [73–79], inspiring the development of future CCIEVIS on the topic.
5. *Attribution*: In recent years, formal methods have been developed to identify changes in the occurrence of adverse health outcomes, and to determine the extent to which those changes may be attributed to climate change [80]. Multi-step attribution, for instance, consists of a) attributing an observed change in a variable of interest to a change in climate or other environmental variable and b) attributing the change in climate to external drivers such as greenhouse gas emissions [81]. The 2020 *Lancet* Countdown report showcased the attribution of 76 extreme weather events to climate change, and the effects that a selection of these have had on the health of the population [32]. Future CCIEVIs could be based on attribution to quantitatively understand how climate change makes extreme events associated with health outcomes more likely.
6. *Embracing the unquantifiable*: There are important aspects of the relationship between climate change and health that cannot be quantified or are very difficult to quantify meaningfully. One example of this is human migration [18]. Local-level studies find worsening food security where people move away from places affected by sea level rise and saltwater intrusion, or adverse psychosocial impacts of disrupted lives, social networks and livelihoods for those on the move in a changing climate. It is extremely challenging, however, to attribute human mobility to climate change (and quantify its magnitude), or to develop a global CCIEVI that can quantify links between climate impacts, human mobility, and health.

## Conclusions

In developing *climate change impacts, exposures, and vulnerability indicators* (CCIEVIs), several key challenges have to be addressed around the idea of representing highly complex systems by a discrete set of quantifiable indicators that are updated annually. One challenge is to find globally relevant indicators, with data available across a sufficiently large number of countries, that also have local relevance.

A hazard, exposure, and vulnerability framework has proven to be an effective structure within the *Lancet* Countdown initiative. Around such a framework, *Lancet* Countdown CCIEVIs have been crafted, based on both data availability and the indicators’ relevance to climate change and human health. Over time these indicators have been improved and added to, as data become available and methodologies are refined. An ongoing challenge is how to incorporate those linkages between climate change and health that are clear and well documented but at the same time difficult to measure, quantify, and fit within this framework.

Together, *Lancet* Countdown CCIEVIs present a compelling visualisation of how climate change is increasingly exposing people to the negative health impacts of climate change, both direct and indirect, across land and water. Ultimately the impact of climate change on human health depends not only on the exposure and vulnerability of populations to climate hazards, but also on the extent to which individuals and countries are able to adapt and build resilience. By unveiling the challenges underpinning the evolution of the climate-health system in time and in space, CCIEVIs can be the pillars of a new public health that safeguards and advances global wellbeing in the decades to come.

## Data Availability

Not applicable.
